# Tissue-Specific Profiling of Oxidative Stress-Associated Transcriptome in a Healthy Mouse Model

**DOI:** 10.3390/ijms19103174

**Published:** 2018-10-15

**Authors:** Jung Min Kim, Hyeong Geug Kim, Chang Gue Son

**Affiliations:** 1 Genoplan Korea, Inc., Seoul 06221, Korea; brant@genoplan.com; 2 Liver and Immunology Research Center, Dunsan Hospital of Daejeon University, Daejeon 301-724, Korea; okskjw04@gmail.com

**Keywords:** oxidative stress, gene expression, transcriptome, tissue-specific variation

## Abstract

Oxidative stress is a common phenomenon and is linked to a wide range of diseases and pathological processes including aging. Tissue-specific variation in redox signaling and cellular responses to oxidative stress may be associated with vulnerability especially to age-related and chronic diseases. In order to provide a basis for tissue-specific difference, we examined the tissue-specific transcriptional features of 101 oxidative stress-associated genes in 10 different tissues and organs of healthy mice under physiological conditions. Microarray analysis results, which were consistent with quantitative polymerase chain reaction (qPCR) results, showed that catalase, Gpx3, and Gpx4 were most highly regulated in the liver, kidney, and testes. We also found the tissue-specific gene expression of SOD1 (liver and kidney), SOD2 (heart and muscle), and SOD3 (lung and kidney). The current results will serve as a reference for animal models and help advance our understanding of tissue-specific variability in oxidative stress-associated pathogenesis.

## 1. Introduction

Each organ and tissue is vulnerable to a variety of toxic agents and carcinogenic exposures. The various disease-associated phenomena that occur in different tissues result from their unique biological environments, which are affected by cellular composition, blood circulation, and the chance of exposure to pathogenic factors [1]. The production of reactive oxygen species (ROS) is unavoidable in all aerobic organisms, which derive their energy from the reduction of oxygen. Oxidative stress is therefore considered one of the key mediators in a wide range of pathological processes including inflammation, vascular disorders, cancer, and aging [2,3].

Aerobic organisms have evolved highly efficient and adaptive antioxidant defense mechanisms, and many reports indicate that antioxidant capacity and susceptibility to oxidative stress are highly tissue-specific [4,5]. Tissue-specific reductions in telomere length after treatment with an oxidative stressor (l-buthionine sulfoximine) are associated with the variable antioxidative capacity of each tissue [6]. Therefore, defining the oxidative stress-related characteristics of multiple organs can help elucidate the mechanisms of oxidative stress-associated pathogenesis in each organ or tissue.

On the other hand, it is known that gene expression varies more considerably across organs than across species and that tissue-specific transcriptome profiling tends to be associated with diseases [7]. There are several studies partially reporting tissue-specific gene expression or protein activity after exposure to toxic agents. For example, one group reported the differential expression of glutathione S-transferase (GST) isoenzymes in the liver, kidney, and testis in diabetic rats after treatment with streptozotocin [8]. Another group found that tissue-dependent toxicity was related to oxidative stress in fish exposed to 2,4-dichlorophenoxyacetic acid orazinphosmethyl [9]. In addition, one group reported a tissue-specific transcriptome resource of six different organs in starfish [10]. To date, however, no study has simultaneously compared the oxidative stress-related transcriptome in multiple organs under physiological condition especially in mice.

In order to provide a valuable resource for tissue-specific oxidative stress as an experimental model organism, the present study investigated the genome-wide expression profile of 10 different tissues/organs in healthy mice and identified tissue-specific features of the oxidative stress-associated transcriptome.

## 2. Results and Discussion

We first verified the internal consistency of 50 data sets (ten different tissues of five different mice) and compared the results from 10 tissues using principal component analysis (PCA), which showed a distinct pattern of clustering by tissue. In total, 9131 genes with SD values <0.3 for all 10 tissues collected from five mice were selected from the initial pool of 28,853 genes. The spleen and thymus, liver and kidney, and heart and muscle were most closely clustered (Supplementary Figure S1A). These transcript patterns were in accordance with the hierarchical clustering (HC) structure, reflecting the known similarity of biological functions between these tissues (Supplementary Figure S1B). In particular, the gene expression profiles of the cerebrum and testes were clearly distinct from those of the remaining tissues in the HC analysis and correlation matrix plot, as well as in PCA. These results are very similar to those of previous studies conducted with human organs [11], which supports the quality of the current transcriptome data and the relevance of these findings to the human context.

**Figure 1:**
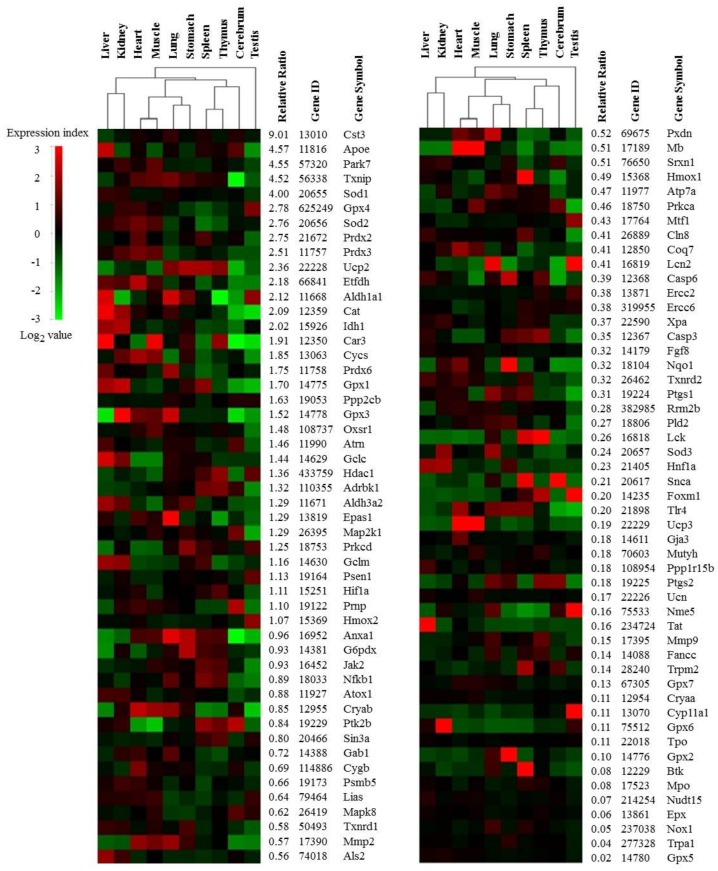
Tissue-specific expression pattern of oxidative stress-related 101 genes. Tissues were ordered according to hierarchical clustering (*x*-axis), while genes were ordered by the relative ratio. The ratio (log base 2) of the signal intensity of each gene within one tissue was divided by the mean signal intensity of all 10 tissues. The red and green colors represent >2-fold up and downregulated genes, respectively.

Identifying the differential transcriptomic features of various organs, tissues, and cell types is important in elucidating the physiology, pathology, and targets of treatments [12]. In the present study, we aimed to investigate the tissue-specific features of the oxidative stress-related transcriptome. We identified 101 oxidative stress-related genes from 9131 genes using the Gene Ontology (GO) database and the search term ‘response to oxidative stress’ (http://www.geneontology.org/). The GO annotations have proven to be remarkably useful for the mining of functional and biological significance from very large datasets, such as microarray results [13]. All of these selected 101 genes showed a 2-fold change in gene expression at least in one tissue compared to the average values across 10 tissues, which illustrates the diverse activities of oxidative stress-related genes in each organ under normal conditions. The HC structure of these genes was slightly different than with the 9131 genes. For example, the clustering of the heart and muscle, spleen and thymus, liver and kidney, and lung and stomach were similar, but the next highest cluster differed between the two sets. The testes and brain tissues had a unique expression profile across the 101 genes compared to the remaining eight tissues, as shown on the heat-map. On the other hand, the cerebrum would be very close to spleen and thymus in the hierarchical clustering (Figure 1 and Supplementary Figure S1B).

Our data reflects known patterns of gene expression across 10 different tissues. In an analysis of big transcriptomics data from human tissues and organs, the testes and brain tissue had the highest number of tissue-enriched protein-coding genes [14]. However, the testis and cerebrum had the lowest transcriptional activity for oxidative stress-related genes (for example catalase) compared to other tissues as our data showed (Figure 1 and Supplementary Table S1). Brain is known to be particularly susceptible to oxidative damage due to its high energy and oxygen demands, its abundance of highly unsaturated fatty acids and its relatively limited antioxidant capacity relative to other organs [15,16]. Testes are also at risk since both spermatogenesis and Leydig cell steroidogenesis are vulnerable to oxidative stress [17]. To compensate for these features of the microenvironment, these two organs have specific blood–tissue barriers, called the blood–testis barrier (BTB) and the blood–brain barrier (BBB), to protect them from immunological processes [18,19].

The production of reactive oxygen species (ROS) is potentially toxic and can impair cellular or tissue integrity [20]. Animals and humans have therefore evolved highly efficient and adaptive antioxidant defense mechanisms, including enzyme-based antioxidants such as superoxide dismutase (SOD), catalase (CAT), and the glutathione (GSH) oxidation/reduction system, and non-enzyme antioxidant molecules [21]. However, oxidative stress and an excessive generation of ROS exceeding the ability of the cell to remove them often occur, and this is linked to a wide range of diseases and pathological processes [22]. The abilities of normal cells to respond to endogenous and exogenous oxidative stress may be compromised by alterations in the expression of antioxidant enzyme genes [23]. To validate the tissue-specific transcriptional profiles (microarray experiments) of mRNA samples isolated from 10 different tissues in healthy mice, we analyzed several well-known or typical genes including *Cat, Gpx3, Gpx4,* and *Sod1* to *3* using quantitative real-time PCR.

**Figure 2:**
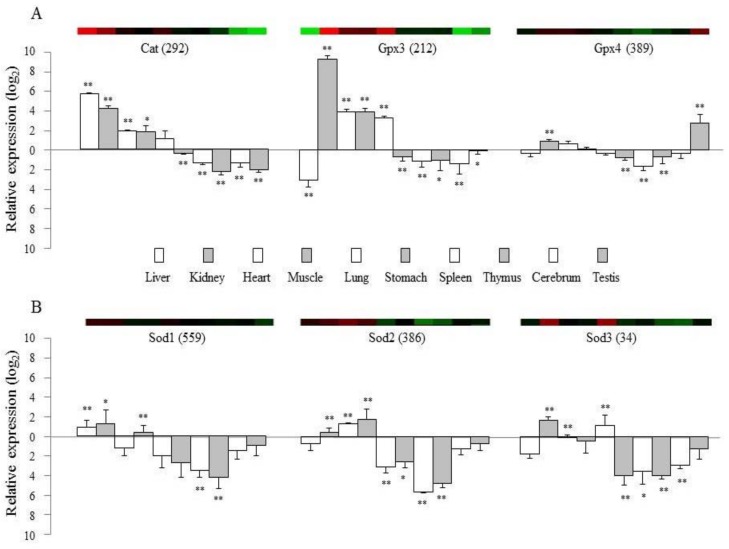
Confirmation of gene expression levels using qRT-PCR. Five mice-derived samples were used for microarray experiment and for qRT-PCR. The top colored panels show the data from microarray analysis, and the bottom graphs present the gene expression results from qRT-PCR for six genes; catalase, catalase (*Cat*), glutathione peroxidase 3 (*Gpx3*), glutathione peroxidase 4 (*Gpx4*), and superoxide dismutase 1 to 3 (*Sod1* to *3*), respectively. The numbers in parentheses indicate the relative transcript levels (the mean signal intensity) of the microarray experiment. Each value represents the mean ± SD. ANOVA indicated *p* < 0.01. The multiple comparisons are not presented; instead, the relative ratio of each tissue gene expression was compared with the mean expression level of rest 10 tissues. The significance was presented as * *p* < 0.05 or ** *p* < 0.01 respectively.

The gene expression patterns from the microarray experiments were mostly consistent with data obtained from quantitative real-time PCR (Figure 2). Moreover, these results reflect available physiological knowledge. The average signal intensities of *Gpx3* and *Gpx4* were very high in our microarray data, and expression of these genes was specific to the kidney and testes. *Gpx3* is known to be synthesized by peroxidase primarily in the kidney and distributed through the bloodstream [24]. Catalase is another important enzyme that catalyzes the removal of hydrogen peroxide from biological tissues, and its gene expression was relatively high in the liver and kidney but low in the testes and brain (Figure 1), which was in accordance with protein activity of catalase (Supplementary Figure S2). This finding correlated with previous analyses of these four organs in rabbit [25] and human tissues [26]. SODs (encoded by the *Sod1* to *Sod3* genes) catalyze the dismutation of two superoxide radicals into hydrogen peroxide and oxygen [27]. These three isoforms of the SOD family are located in the cytoplasm (*Sod1* as Cu/Zn superoxide dismutase), mitochondria (*Sod2* as manganese superoxide dismutase), and extracellular space (*Sod3*), respectively [28]. *Sod1* showed the highest gene expression levels in the liver and kidney, whereas *Sod2* was most highly expressed in the heart and muscle in both the microarray and qPCR analyses (Figure 2). Mice deficient in *Sod1* have an accelerated accumulation of mutations especially in the liver and kidney [29]. The elevated level of ROS is known to contribute considerably to the deterioration of cardiac function [30]. The function of *Sod2* was identified based on a lethal cardiomyopathy in *Sod2*-knockout mice [31] and an association with nonfamilial idiopathic dilated cardiomyopathy in Japanese population [32]. In contrast to intracellular *Sod1* and *Sod2*, the expression of *Sod3* was known to be restricted to several tissues, mainly in the lung and kidney [33,34], which is exactly consistent with both our microarray and qPCR results (Figure 2).

These results show a difference in the transcription of these enzyme-based antioxidants across 10 tissues. Analysis of six antioxidation-related genes in two classically immune-related organs, the spleen and thymus, showed the lowest level of gene activity compared to other organs in our study (Figure 2), and these organs are known to have high levels of endogenous melatonin as an antioxidant agent [35]. The testes have a low oxygen tension to minimize the risk of free radical-mediated damage [17], and our study showed the lowest level of gene activity in the testes for the 101 genes (Figure 1 and Supplementary Table S1). In particular, *Gpx4* and *Cyp11a1* were the most and least highly expressed genes, respectively, in the testes but reversely in cerebrum (Figure 1 and Figure 2). *Gpx4* gene serves dual functions in normal sperm development and gonadal development as well as in protection of the cell membrane against lipid peroxidation [36]. Besides involving in corticosteroid synthesis, *Cyp11a1* is known to mediate the metabolism of vitamin D2, a membrane antioxidant [37].

It is known that gene expression varies more considerably across organs than across species and that tissue-specifically expressed genes tend to be associated with the tissue-associated diseases [7]. One study presented a similarity in the RNA-seq data-derived transcriptome across tissues using three male mice and three male rats [38]. Furthermore, we compared the pattern of the transcriptome between our data and the RNA-seq platform using enzyme-based antioxidant genes including *CAT* and isotypes of *SOD* and *GSH peroxidase*, respectively. We confirmed the parallel feature of them (Figure S3).

Our data on gene expression of oxidative stress-related genes in healthy mice are consistent with data previously reported in two human databases, TiGER (Tissue-specific Gene Expression and Regulation) [39] and TiSGeD (Tissue-Specific Genes Database) [40]. Genome-wide analyses support the association between tissue-specific gene expression and tissue-restricted protein function, and our data also correlate generally with human protein expression data [41]. However, our data are limited as they are derived from mice, and lack sex-related and age-related information. Nevertheless, our animal-based strategy has the advantage that it allowed us to study the molecular underpinnings of tissue-specific oxidative stress and associated disorders. In addition, we have released our raw dataset to GEO as reference data (https://www.ncbi.nlm.nih.gov/geo/query/acc.cgi?acc=GSE111159) for further analyses.

Taken together, we found that the oxidative stress-related transcriptome in 10 organs and tissues reflects their physiological roles, as a notably tissue/organ-specific manner, most differently in testes. The information obtained from this study will be helpful in advancing our molecular understanding of tissue- and organ-specific oxidative stress-associated effects and disorders. The present study paves way for an animal model of tissue-specific variation in oxidative stress-associated pathogenesis.

## 3. Materials and Methods

### 3.1. Mouse Tissue Samples and RNA Extraction

Five male BALB/c mice (six weeks old) were purchased from a commercial animal breeder (OrientBio, Seongnam, Korea). The mice were housed in an auto-controlled pathogen-free animal room at 22 ± 2 °C under a 12:12 h light/dark cycle, and they were provided with commercial pellets and tap water *ad libitum* for five weeks. The mice were sacrificed by complete blood collection via the inferior vena cava under anesthesia with ether. The spleen, liver, left kidney, left testis, stomach, thymus, lung, heart, brain, and left rectus femorismuscle were removed orderly and then stored in RNAlater solution (Ambion, Austin, TX, USA) at −20 °C. Experiments were designed and performed strictlyin accordance with the Guide for the Care and Use of Laboratory Animals (8th edition, NIH update 2011) and approved by the Institutional Animal Care and Use Committee of Daejeon University (Animal ethical clearance number: DJUARB 2016-034 to 6, 03 March 2016).

Total RNA was extracted using the RNeasy midi kit (Qiagen, Valencia, CA, USA) according to the manufacturer’s instructions. RNA quality and concentration were assessed using an Agilent Bioanalyzer 2100 (Agilent Technologies, Santa Clara, CA, USA) and a NanoDrop ND-1000 spectrophotometer (NanoDrop Technologies, Wilmington, DE, USA), respectively. The absorbance ratio at 260:280 nm for all samples was >1.8 and the RIN value was >8. The integrity of RNA samples was also ascertained by the presence of distinct 28S and 18S ribosomal RNA bands in agarose gels after electrophoretic resolution. Five mouse-derived samples were used for both microarray experiments and verification of gene expression using quantitative real-time PCR (qRT-PCR).

### 3.2. Microarray Experiments and Data Collection

Microarray analysis was performed for each tissue sample using the GeneChip Mouse gene 1.0 ST array according to the manufacturer’s protocol (Affymetrix, Santa Clara, CA, USA). Briefly, cDNA was synthesized from total RNA (100 ng) using a T7-oligo(dT) primerand Superscript RT II kit (Invitrogen, Carlsbad, CA, USA), and cDNA was purified with a QIAquick PCR Purification Kit. Next, biotin-labeled cRNA was synthesized using an Affymetrix RNA transcription labeling kit (Affymetrix). After cleaning and fragmentation, a total of fifty cDNA samples from each of 10 different tissues collected from the five mice were hybridized using the Mouse gene 1.0 ST array (which contains 28,853 genes) for 16 h. After washing and staining with streptavidin phycoerythrin solution and antibody solution, images were obtained by capturing the fluorescence intensity using a GMS 418 Array Scanner (Affymetrix). Microarray data were uploaded using GenPlex^TM^ v3.0 (Istech Inc., Seoul, Korea) and GeneSpring GX 7.3 software (Agilent Technology) and normalized with RMA [42]. Further statistical analyses were performed primarily using tools included in GenPlex and GeneSpring GX 7.3. Functional annotation of genes was performed per the Gene Ontology™ Consortium (http://www.geneontology.org/index.shtml). Gene classification was based on searches using the GeneCards (http://www.genecards.org/), BioCarta (http://www.biocarta.com/), DAVID (http://david.abcc.ncifcrf.gov/), and Medline (http://www.ncbi.nlm.nih.gov/) databases.

### 3.3. Confirmation of Tissue Gene Expression Homogeneity

Based on the whole-transcript expression of 28,853 genes, we selected 9131 genes that met two conditions: more than 2-fold change in at least one tissue and SD values <0.3 estimated from the five individual samples for all 10 different tissues. The selected genes were considered to constitute a data set of reliable genes. Principal component analysis (PCA) was used to cluster the signal intensity values of all 50 data sets generated for the 9131 genes using GenPlex software. A hierarchical clustering (HC) algorithm was used to group tissues based on similar expression patterns using the Euclidean distance metric and the same gene lists. The correlation representation was derived from an exploded correlation matrix plot using tools included in GenPlex.

### 3.4. Selection and Comparative Analysis of Oxidative Stress-Associated Genes in 10 Tissues

Genes related to oxidative stress were selected using the Gene Ontology (GO) database with the search term ‘response to oxidative stress’ (http://www.geneontology.org/). A total of 101 genes met the selection criterion of a 2-fold change in gene expression in at least one tissue compared to average values across 10 tissues (Listed in Supplementary Table S1). The ratio (log base 2) of the signal intensity of each tissue was divided by the mean signal intensity of all 10 tissues. Tissues were ranked according to the HC and genes were ranked based on the relative ratio. The HC was represented using tools provided by GenPlex (the Euclidean distance metric).Next, the mean signal intensity of each gene was determined in all 10 tissues. Finally, the relative ratio of the mean signal intensity was computed for all 10 tissues. The relative ratio was calculated by dividing the mean signal intensities across all the genes (value = 140.1).

### 3.5. Confirmation Using qRT-PCR

We used qRT-PCR (SYBR green method) to verify and quantify the changes in expression of some of the genes identified by microarray analysis. Briefly, we synthesized cDNA from 1 μg total RNA using reverse transcriptase according to the manufacturer’s instructions (Invitrogen, Carlsbad, CA, USA). PCR was performed for seven genes including β-actin on ABI Prism^®^ 7900HT Sequence Detection System (PE Applied Biosystems, Foster City, CA, USA). Each reaction was performed in triplicate under thermal cycling conditions as follow; 95 °C for 10 min followed by 45 cycles of 95 °C for 10 s, 60 °C for 15 s, and 72 °C for 20 s. The relative transcript levels of genes were determined by comparing to β-actin as the endogenous control. The sequences of the forward and reverse primers (http://bioinfo.ut.ee/primer3-0.4.0/) were as follows: superoxide dismutase 1, soluble (Sod1; NM_011434) (forward) 5′-CAG AAG GCA AGC GGT GAA C-3′ and (reverse) 5′-CAG CCT TGT GTA TTG TCC CCA TA-3′; Sod2 (mitochondrial; NM_013671) (forward) 5′-CCC AGA CCT GCC TTA CGA CTA T-3′ and (reverse) 5′-GGT GGC GTT GAG ATT GTT GA-3′; Sod3 (extracellular; NM_011435) (forward) 5′-AAA GGT TCC CAA ATA CTC TCT CTA AGG-3′ and (reverse) 5′-CCC ACC CCC AAG TTC CAT-3′; catalase (Cat; NM_009804) (forward) 5′-GGA CGC TCA GCT TTT CAT TC-3′ and (reverse) 5′-TTG TCC AGA AGA GCC TGG AT-3′; glutathione peroxidase 3 (Gpx3; NM_001083929) (forward) 5′-GAT GTG AAC GGG GAG AAA GA-3′ and (reverse) 5′-TTC ATG GGT TCC CAA AAG AG-3′; glutathione peroxidase 4 (Gpx4; NM_008164) (forward) 5′-TAA GAA CGG CTG CGT GGT-3′ and (reverse) 5′-GTA GGG GCA CAC ACT TGT AGG-3′; and β-actin (Actb; NM_007393) (forward) 5′-TTG CTG ACA GGA TGC AGA AG-3′ and (reverse) 5′-GTA CTT GCG CTC AGG AGG AG-3′.

### 3.6. Statistical Analyses

The results obtained from the qRT-PCR and catalase activity analyses were expressed as the mean ± standard deviation (SD). Significant differences between groups were evaluated by one-way analysis of variance (ANOVA) followed by post-hoc multiple comparisons using IBM SPSS statistics software, ver. 20.0 (SPSS Inc., Chicago, IL, USA). Differences with *p* < 0.05 or *p* < 0.01 were considered statistically significant.

## Supplementary Materials

Supplementary materials can be found at http://www.mdpi.com/1422-0067/19/10/3174/s1.

## Abbreviations

BBB	blood–brain barrier
BTB	blood–testis barrier
Cat	catalase
GO	gene ontology
GSH	glutathione
Gpx3	glutathione peroxidase 3
Gpx4	glutathione peroxidase 4
HC	hierarchical clustering
PCA	principal component analysis
ROS	reactive oxygen species
Sod1	superoxide dismutase 1
Sod2	superoxide dismutase 2
Sod3	superoxide dismutase 3
