# Supplementary material for: Tissue-Specific Profiling of Oxidative Stress-Associated Transcriptome in a Healthy Mouse Model
Source: Int J Mol Sci. 2018 Oct 15;19(10):3174. doi: 10.3390/ijms19103174 (PMC6214011; doi:10.3390/ijms19103174)
Supplement: Supplementary file 1 [file ijms-19-03174-s001.pdf]

Supplementary Materials

Tissue-Specific Profiling of Oxidative Stress-Associated Transcriptome in a Healthy Mouse Model

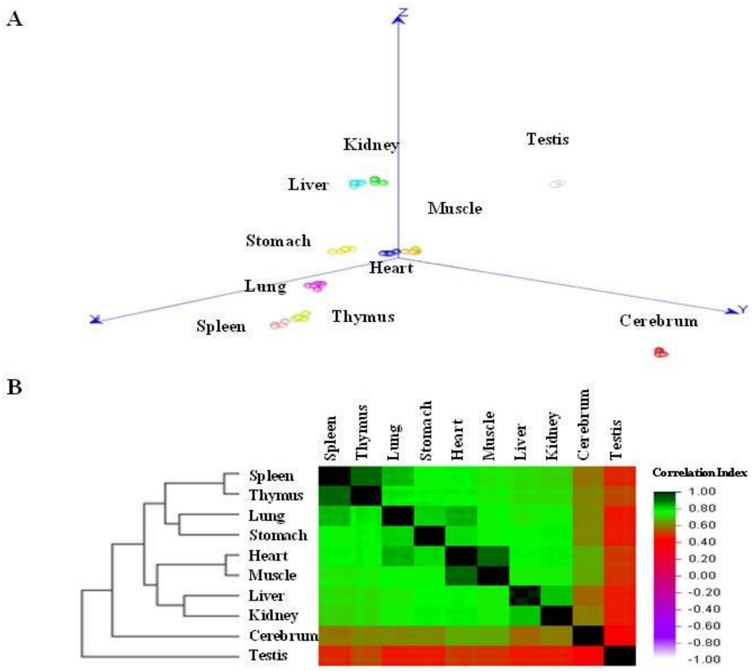

Figure S1. Principal component analysis and correlation matrix. PCA (**A**) shows the internal consistency and relationship of 50 data sets while HC structure and correlation matrix plot (**B**) represent the gene expression similarity among 10 different tissues using 9131 genes. In the correlation matrix plot, black squares represent highest similarities, and red squares represent low similarities. The PCA and correlation matrix plot were represented using tools provided by GenPlex and GeneSpring GX 7.3 software.

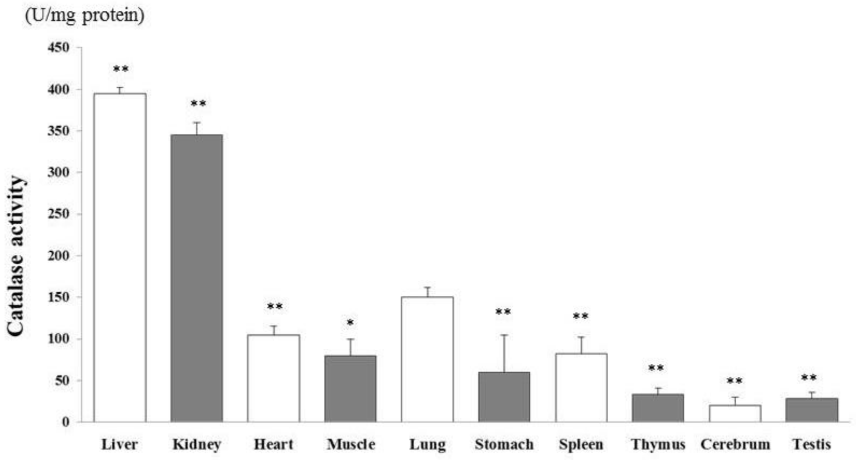

Figure S2. Compassion of catalase activity among 10 tissues. Catalase activities from total 50 tissues of 10 different organs were analyzed. The activity was represented by measuring the capacity to decompose hydrogen peroxide to water and oxygen.

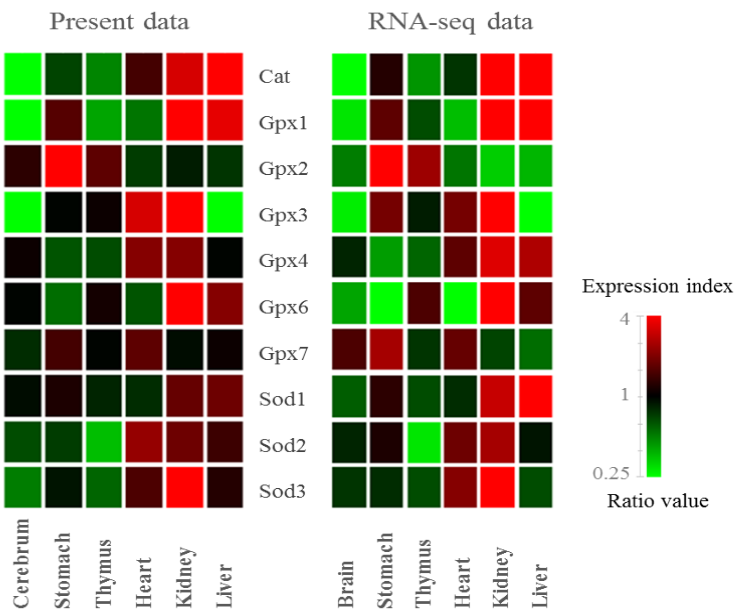

Figure S3. Compassion with public data. The current microarray data were compared with RNA-seq data available in the public [38]. This comparison was performed to the well-known 10 genes for oxidative enzymes in only 6 organs which were contained in both our data and the public data. Clustering analyses were performed using the GeneSpring GX 7.3. The ratio of the signal intensity of each gene within one tissue was divided by the mean signal intensity of all 6 tissues. The red and green colors represent 2-fold up and down-regulated genes, respectively.

Table S1. Tissue-specific fashion of oxidative stress-related genes in different tissues.

Gene ID	Gene symbol	[a]Ratio of the signal intensity of each tissue (log base 2)										[b]Exp. level	[c]Ratio signal/ mean
		Li	Ki	He	Mu	Lu	St	Sp	Th	Ce	Te		
110355	Adrbk1	-0.76	-0.66	-0.34	-0.08	0.22	-0.05	1.28	1.41	0.63	-1.64	184.8	1.32
11668	Aldh1a1	2.56	-2.13	-0.52	0.29	2.44	1.18	-0.18	-3.50 [e]	-1.80	1.66	297.3	2.12
11671	Aldh3a2	1.81	1.15	-0.52	-1.24	0.45	1.08	-0.62	-0.09	-0.95	-1.08	180.3	1.29
74018	Als2	1.70 [d]	0.71	-0.46	-0.01	-0.42	-0.40	-0.53	-0.35	0.12	-0.37	78.8	0.56
16952	Anxa1	-1.59	-1.12	0.81	0.90	2.65	2.07	0.91	0.87	-3.35[e]	-2.13	133.8	0.96
11816	Apoe	2.27	-0.95	0.16	-0.96	0.15	-0.52	0.37	0.20	0.96	-1.70	640.0	4.57
11927	Atox1	0.78	0.76	0.13	-0.45	-0.12	0.25	0.08	-0.06	-0.88	-0.48	123.5	0.88
11977	Atp7a	-0.76	0.40	-0.50	-0.32	1.20	0.79	0.23	0.35	-1.05	-0.35	65.7	0.47
11990	Atrn	0.86	0.02	-0.20	-0.44	0.35	0.36	-0.51	-0.35	0.95	-1.04 [d]	203.8	1.46
12229	Btk	-0.19	-0.75	-0.50	-0.51	0.67	-0.33	3.41 [d,e]	-0.07	-0.74	-0.99	11.6	0.08
12350	Car3	3.37 [e]	-0.07	-1.36	2.82	-0.17	0.69	-1.34	1.72	-3.03[e]	-2.62	268.0	1.91
12367	Casp3	0.48	-0.12	-0.93	-0.97	0.00	0.94	1.15	1.56	-0.65	-1.47	48.9	0.35
12368	Casp6	0.71	0.21	-0.35	-1.49	0.41	2.23	-0.02	1.03	-1.94	-0.80	54.3	0.39
12359	Cat	2.81	1.76	0.38	0.05	0.69	-0.21	-0.06	-0.59	-2.21	-2.62	292.9	2.09
26889	Cln8	0.58	0.14	0.09	0.12	-0.07	0.08	1.09	-0.26	-0.03	-1.74	57.5	0.41
12850	Coq7	0.02	0.51	1.72	0.99	-0.62	0.19	-1.14	-0.47	-0.41	-0.78	57.9	0.41
12954	Cryaa	0.09	0.09	0.10	0.27	-0.23	-0.13	-0.11	0.03	-0.06	-0.05	15.8[g]	0.11
12955	Cryab	-1.58	0.41	2.22	1.81	1.65	0.41	-1.53	-1.38	0.33	-2.34	118.9	0.85
13010	Cst3[f]	-0.74	-0.23	0.24	0.09	0.56	-0.11	0.31	-0.23	0.38	-0.27	1,261.0	9.01
13063	Cycs	-0.22	1.08	1.88	1.49	-1.35	0.53	-1.03	-0.46	-0.21	-1.70	259.2	1.85
114886	Cygb	-0.19	-0.55	1.41[d]	0.25	0.13	0.22	-0.52	-0.46	0.34	-0.64	96.5	0.69
13070	Cyp11a1	-0.57	-0.60	-0.74	-0.42	-0.84	-0.47	-0.52	-0.03	-0.22	4.41 [d,e]	15.3	0.11
13819	Epas1	0.45	-0.47	0.83	0.41	2.91	-0.59	-0.14	-1.80	-0.42	-1.19	180.8	1.29
13861	Epx	0.16	0.04	0.01	0.12	-0.19	-0.14	-0.03	0.17	-0.05	-0.11	7.7	0.06
13871	Ercc2	-0.17	-0.20	-0.19	-0.10	-0.09	-0.23	-0.11	0.45	-0.17	0.81	53.4	0.38
319955	Ercc6	0.07	-0.02	-0.62	-0.45	-0.03	-0.21	0.42	0.27	0.21	0.36	52.5	0.38
66841	Etfdh	1.15	0.92	2.11	0.78	-0.68	0.17	-1.13	-0.27	-1.55	-1.48	305.5	2.18
14088	Fancc	-0.46	-0.32	0.03	-0.44	0.67	-0.46	0.41	0.72	-0.51	0.36	19.9	0.14
14179	Fgf8	0.32	0.07	0.12	0.19	-0.22	0.09	-0.16	-0.19	-0.05	-0.17	44.8	0.32
14235	Foxm1	-0.92	-1.01	-0.88	-0.95	-0.72	-0.05	0.70	2.05	-1.21	2.97	27.6	0.20
14381	G6pdx	-1.68	0.25	-1.08	-0.31	0.42	2.09	0.78	0.57	-0.42	-0.64	129.8	0.93
14388	Gab1	-0.42	0.48	0.66	0.00	0.99	-0.01	-0.61	-0.89	-0.14	-0.05	100.7	0.72
14629	Gclc	2.49	1.37	-1.50	-1.52	0.46	0.24	0.24	-0.62	-0.56	-0.59	201.5	1.44
14630	Gclm	1.78	1.61	-0.83	-0.71	-0.60	0.31	0.78	-1.20	-0.72	-0.43	162.3	1.16
14611	Gja3	0.02	-0.11	1.13 [d]	-0.07	-0.12	-0.13	-0.32	-0.06	-0.05	-0.29	25.2	0.18
14775	Gpx1	1.91	2.25	-0.50	-0.76	-0.17	0.57	1.58	-0.81	-1.85	-2.20	238.2	1.70
14776	Gpx2	-0.77	-0.65	-0.81	-0.49	0.76	4.07 [d,e]	-0.91	-0.08	-0.42	-0.71	14.3	0.10
14778	Gpx3	-2.71	3.72 [e]	1.22	1.08	2.37	-0.44	-0.46	-0.41	-2.58	-1.78	212.3	1.52
625249	Gpx4	-0.24	0.62	0.65	0.23	-0.19	-0.64	-1.05	-0.59	-0.21	1.43	389.1	2.78
14780	Gpx5	0.20	0.28	0.12	0.10	-0.11	-0.16	-0.09	-0.06	-0.01	-0.26	3.1	0.02

75512	Gpx6	0.34	4.81 [e]	-0.89	-0.81	-1.04	-1.02	-0.99	-0.46	-0.51	0.57	15.7	0.11
67305	Gpx7	-0.08	-0.15	0.39	0.37	0.27	0.17	-0.34	-0.12	-0.33	-0.17	18.9	0.13
433759	Hdac1	-0.81	-0.30	-0.59	-1.10	0.41	0.21	0.76	1.66	-1.31	1.08	190.1	1.36
15251	Hif1a	-0.21	0.17	0.37	-0.66	0.03	0.01	0.38	0.94	-0.08	-0.95	156.1	1.11
15368	Hmox1	0.28	0.09	-0.49	-0.90	0.11	0.34	3.22 [e]	-0.66	-1.62	-0.39	68.5	0.49
15369	Hmox2	-0.14	-0.11	0.18	-0.29	-0.44	-0.30	-0.28	0.06	-0.33	1.63 [d]	149.8	1.07
21405	Hnf1a	1.76	1.91	-0.55	-0.39	-0.73	0.80	-0.71	-0.52	-0.78	-0.79	31.9	0.23
15926	Idh1	1.79	2.04	-0.23	-0.62	-0.25	0.32	-0.84	-0.74	-1.36	-0.11	282.7	2.02
16452	Jak2	-0.51	-0.30	-0.24	0.48	0.31	0.13	1.10	0.89	-0.27	-1.60	130.2	0.93
16818	Lck	-1.23	-1.18	-1.33	-1.18	0.54	-0.84	2.52	5.25 [e]	-1.37	-1.18	35.8	0.26
16819	Lcn2	-0.03	-0.79	-0.66	-1.24	2.77	-1.68	-0.63	-0.40	-1.99	4.65[e]	57.7	0.41
79464	Lias[f]	0.41	0.41	0.53	0.63	-0.72	-0.47	-0.66	-0.20	-0.52	0.58	89.3	0.64
26395	Map2k1	-0.02	-0.28	-0.08	0.51	-0.01	0.54	0.15	-0.13	1.29	-1.99	180.1	1.29
26419	Mapk8	-0.16	-0.27	0.06	0.46	-0.61	-0.68	-0.30	0.05	0.97	0.48	87.3	0.62
17189	Mb	-1.38	-1.41	4.80 [e]	3.80 [e]	-0.22	-0.59	-1.58	-0.50	-1.42	-1.50	71.2	0.51
17390	Mmp2	-1.09	-0.98	1.67	1.31	1.73	0.64	-0.26	-0.03	-1.52	-1.47	79.6	0.57
17395	Mmp9	-0.42	-0.41	-0.11	0.14	0.76	-0.04	0.20	1.08 [d]	-0.41	-0.78	21.5	0.15
17523	Mpo	0.17	0.03	-0.32	0.08	-0.28	-0.19	0.46	0.31	0.02	-0.27	11.7 [g]	0.08
17764	Mtf1	0.03	-0.47	-0.49	-0.13	-0.20	-0.23	0.07	-0.08	-0.19	1.69 [d]	60.3	0.43
70603	Mutyh	-0.18	-0.36	-0.10	0.53	-0.20	-0.11	0.10	0.52	-0.19	-0.01	24.7	0.18
18033	Nfkb1	-0.16	-0.66	0.10	-0.11	1.03	0.09	1.38	0.97	-1.17	-1.46	124.2	0.89
75533	Nme5	-1.02	0.33	-0.22	-0.34	1.04	-1.24	-1.75	-1.48	0.62	4.06[e]	22.5	0.16
237038	Nox1	-0.07	-0.04	-0.25	-0.12	0.71	-0.20	0.38	0.06	-0.17	-0.31	7.6	0.05
18104	Nqo1	-1.12	0.63	1.29	0.21	-0.86	4.20 [e]	-0.96	-0.05	-1.71	-1.62	44.8	0.32
214254	Nudt15	0.40	-0.07	0.17	0.19	-0.11	-0.25	-0.09	-0.10	0.11	-0.26	9.1	0.07
108737	Oxsr1	-0.19	-0.17	0.47	1.02	0.23	0.10	-0.13	0.27	-0.18	-1.41	207.1	1.48
57320	Park7	0.12	0.70	0.24	1.08 [d]	-0.30	-0.40	-0.48	0.10	-0.27	-0.78	637.2	4.55
18806	Pld2	-0.03	-0.29	0.26	0.44	0.73	0.95	-0.13	-0.64	0.08	-1.38 [d]	37.6	0.27
108954	Ppp1r15b	0.76	-0.02	-0.32	0.07	-0.30	0.21	0.10	0.04	-0.31	-0.23	24.5	0.18
19053	Ppp2cb	-0.26	-0.16	-0.23	-0.22	0.37	0.13	-0.42	0.01	0.40	0.38	228.3	1.63
21672	Prdx2	-0.29	0.01	1.01	-0.02	-0.51	0.16	0.87	-0.19	0.15	-1.19	385.6	2.75
11757	Prdx3	0.32	0.89	1.33	1.08	-0.85	-0.42	-0.13	-0.34	-0.94	-0.93	351.7	2.51
11758	Prdx6	1.17	0.02	0.19	-0.45	1.77	-0.41	-1.15	-0.76	-0.04	-0.35	244.9	1.75
18750	Prkca	-0.61	-0.75	0.06	1.24	0.26	0.14	0.24	0.32	1.15	-2.05	64.4	0.46
18753	Prkcd	-1.33	0.12	-1.15	-1.06	0.31	1.56	0.65	0.31	-0.26	0.85	174.7	1.25
19122	Prnp	-0.97	0.37	0.71	0.49	0.25	0.00	-0.73	-0.76	2.02	-1.37	154.1	1.10
19164	Psen1	-0.35	0.21	-0.44	-0.42	-0.03	0.17	0.00	0.14	-0.38	1.10[d]	158.0	1.13
19173	Psmb5	0.28	0.72	0.57	0.17	-0.46	0.08	-0.79	-0.05	-0.12	-0.41	93.1	0.66
19224	Ptgs1	0.16	0.40	0.38	-1.00	1.55	0.78	1.15	-0.69	-0.81	-1.93	42.7	0.31
19225	Ptgs2	-0.96	-0.73	0.14	-0.75	1.18	0.76	-1.15	1.46	1.49	-1.43	24.7	0.18
19229	Ptk2b	0.27	0.53	-1.80	-2.32	-0.36	-0.19	1.66	1.24	2.14	-1.17	117.8	0.84
69675	Pxdn	-0.38	-0.38	1.23	0.75	2.30	-0.12	-1.21	-1.03	-0.12	-1.04	73.1	0.52
382985	Rrm2b	-0.89	0.37	0.53	0.34	0.41	-0.24	0.23	0.07	0.07	-0.89	39.1	0.28

20466	Sin3a	-0.22	-0.40	-0.32	-0.47	0.14	-0.14	0.64	0.96	-0.60	0.40	112.4	0.80
20617	Snca	-1.00	-0.59	-0.51	-1.03	0.47	-0.89	2.80	-0.98	2.99	-1.26	29.0	0.21
20655	Sod1	0.60	0.50	-0.21	-0.21	0.27	0.01	-0.09	-0.19	-0.08	-0.60	559.4	4.00
20656	Sod2	0.45	0.86	1.27	0.88	-0.70	-0.06	-1.34	-0.95	-0.15	-0.24	386.5	2.76
20657	Sod3	-0.27	1.63	-0.04	-0.20	1.73	-0.48	-0.23	-0.90	-1.04	-0.20	33.9	0.24
76650	Srxn1	0.42	0.58	-0.01	0.08	-0.39	0.39	-0.96	-0.26	0.70	-0.55	71.0	0.51
234724	Tat	6.27 [d,e]	-0.92	-0.05	-0.45	-0.82	-0.86	-0.92	-0.68	-0.80	-0.77	22.7	0.16
21898	Tlr4	-0.94	-0.58	1.74	0.05	1.27	1.40	1.41	-0.31	-1.90	-2.16	28.1	0.20
22018	Tpo	0.11	-0.02	-0.16	-0.01	-0.10	-0.01	0.02	0.01	0.05	0.10	15.9	0.11
277328	Trpa1	0.05	0.07	-0.14	0.02	-0.13	0.47	-0.10	0.00	0.04	-0.28	5.3	0.04
28240	Trpm2	-0.01	-0.45	-0.64	-0.34	-0.20	-0.49	1.88 [d]	0.04	0.86	-0.65	19.6	0.14
56338	Txnip	-0.70	0.22	0.96	1.12	1.33	0.77	0.45	0.35	-3.48 [e]	-1.04	632.7	4.52
50493	Txnrd1	0.60	0.50	-0.17	0.37	-0.12	0.82	-0.42	-0.04	-0.48	-1.06 [d]	81.6	0.58
26462	Txnrd2	0.79	0.34	0.76	0.33	-0.39	-0.28	0.70	-0.65	-0.48	-1.12 [d]	45.2	0.32
22226	Ucn	0.23	-0.01	0.00	0.38	-0.15	-0.10	-0.12	-0.11	-0.01	-0.11	23.8	0.17
22228	Ucp2	-1.52	-0.54	-0.36	-1.25	1.42	1.97	1.92	1.56	-2.15	-1.06	330.7	2.36
22229	Ucp3	-1.07	-0.99	3.11 [e]	3.66 [e]	-0.50	-1.04	-0.83	-0.27	-0.99	-1.07	27.1	0.19
22590	Xpa	0.30	0.61	0.00	-0.31	-0.02	-0.16	0.49	0.29	-0.62	-0.57	52.0	0.37

[a] The ratio (log base 2) of each tissue signal intensity was divided by the mean signal intensity of all 10 tissues. [b] The mean signal intensity of all 10 tissues in each gene. [c] The ratio of the mean signal intensity of all 10 tissues in each gene was divided by the mean value of 101 genes (value = 140). [d] An absolutely tissue-specific fashion, cut-off > 2-fold only one tissue. [e] The tissue-specific fashion, cut-off > 8-fold. Abbreviations are as follows: Sp, spleen; Th, thymus; Lu, lung; St, stomach; He, heart; Mu, muscle; Li, liver; Ki, kidney; Ce, cerebrum; and Te, testis.
